# Supplementary material for: Benford’s Law applies to word frequency rank in English, German, French, Spanish, and Italian
Source: PLoS One. 2023 Sep 14;18(9):e0291337. doi: 10.1371/journal.pone.0291337 (PMC10501622; doi:10.1371/journal.pone.0291337)
Supplement: Attachment — Submitted filename: Reviewer report PONE-D-23-08610.pdf [file pone.0291337.s001.pdf]

PONE-D-23-08610

Reviewer comments

The present paper demonstrates that Benford's Law applies to ranked word frequencies in English, German, French, Spanish, and Italian. The author(s) computed the word frequency ranks using the Google Ngram Viewer corpus to prove this. By examining the first significant digit of the frequency ranks, the author(s) shows that the distribution of frequencies adheres to the expected pattern outlined by Benford's Law. Furthermore, across various sources such as news, books, and social media the author(s) investigated, we consistently found conformity to Benford's Law. Moreover, the authors analysed users' posts on social media and discovered that the vast majority of collected posts followed Benford's Law, while significant deviations from this pattern often indicated the presence of spam bots.

**Introduction**

In referring to Benford's law studies in general and specifically on the stock market, more recent works may help contextualise. For example, Ausloos, M., Ficcadenti, V., Dhesi, G., & Shakeel, M. (2021). Benford's laws tests on S&P500 daily closing values and the corresponding daily log-returns both point to huge non-conformity. Physica A: Statistical Mechanics and its Applications, 574, 125969.

The following statement should be simplified in favour of readers not familiar with Google 1-gram corpora. Furthermore, it is unclear what is intended by word's frequency rank; namely, have you applied Benford's law on the ranks or the frequencies? It may become clearer later in the text, but I feel it is important to say it here. Finally, what is "gold standard" in this context?

"We conducted an analysis of language use in a variety of corpra. To determine a word's frequency rank (e.g. what is the 314th most popular word in English), we measured frequency using the Google 1-gram corpra (reviewer's note: CORRECT THE TYPO HERE) for English, German, French, 13 Spanish, and Italian. These frequency ranks were used as the gold standard rank in each language."

A similar comment on the clarity of what "rank" and what "frequency" are under investigation should be applied to the paragraph related to social media posts.

**Background and Related Work**

This section could be improved by adding a practical example of the type of analysis that can be done with Benford's law in linguistics. For example, mentioning a word, its rank in a corpus, and the way to consider its frequency. This comment is connected with the one made above about clarity.

**Establishing Word Frequency Rank**

One reads, "Google makes frequency counts available for each word. To calculate frequency and frequency rank, we first removed tokens that were not words (numbers, punctuation) and then sorted by frequency. We assigned each word a frequency rank beginning at 1 for the most popular word (e.g. "the" in the English corpus)." taken that a corpus is a book and that a corpora is a collection of books in a given language, does one have a rank for each word in every corpus? So, for example, one has N English books, each having a number of words and associated frequencies. I understand from the Results sections that words are considered altogether. Namely, one gets "approximately 1 trillion words" with their frequencies and ranks for English, correct? If so, I suggest to clarify here.

**Additional Corpra**

When the author(s) starts this section, the analysis and its results are not presented yet, but the basement of the sub-section is a need to validate the results. This is understandable, but a more thoughtful discussion of the potential results and their generalisability is necessary before introducing a “validation” argument.

In the following statement, “If Benford's Law applies robustly to language use, it should be observable in many contexts, corpra of many sizes, and both balanced and unbalanced corpra.” what does “balanced” mean?

**Results**

In this section, one finally understands, with an example, the concept of “frequency rank”. The usage of this approach is partially justified in the section “Rank-frequency distribution, Zipf's Law, and Language” but it is not enough because the obvious thought I had by reading is “why not use the frequencies directly?” I like the idea of using the ranking, so I am not discouraging, but some more on the reasons, the benefit of it, might be beneficial.

**User-Level Analysis on Social Media**
The methodology here must be clarified. Is the analysis based again on the “frequency rank”?

I do not get if the conclusion related to the fact that “bot” social network accounts are less Benford’s law compliant comes from a visual inspection or if it is possible to have some statistical test to validate that point.

**Conclusions:**
If the author(s) decides to keep any of the comments on board, please revise the conclusion accordingly. Furthermore, I suggest that, given the quality of the results in the field, more academically informed conclusive remarks can be given on the areas covered: the linguistic aspect and the social network component.

**General comments:**
When one removes non-standard text, I refer, e.g. to “Other non-standard words are unlikely to appear in the Google corpra and will be ignored” there might be a “manipulation” of the data that somewhat connect to what is done in the case of accountancy when some figures are systematically changed. Therefore, extra care should be used. Some of those words might be neologisms, words that are outside current dictionaries but in use in “hurban” conversation; this is especially relevant for the component of the analysis related to social networks. My question would be, does consider words in official dictionaries or including those not-in-dictionaries would change the results drastically? If it is not possible to re-do the analysis, I understand. I am not necessarily asking for that, but a few comments on the impact on Benford’s law validity would help. This is partially there already when the %s are reported, but a clear statement where one says that these numbers are not enough for breaking Benford’s law may reassure a reader. For example in “Ausloos, M., Ficcadenti, V., Dhesi, G., & Shakeel, M. (2021). Benford’s laws tests on S&P500 daily closing values and the corresponding daily log-returns both point to huge non-conformity. Physica A: Statistical Mechanics

and its Applications, 574, 125969” a point regarding the inclusion or exclusion of the “zeros” from the returns analysis is made when evaluating the second digit law.

Check the usage of the words “corpora” and “corpus” I feel sometimes the usage of the words is interchanged, but the meaning is different.

Congratulations on using genuine and non-genuine accounts for the analysis related to the bots! I really like it!
